# Hepatocyte Nuclear Factor 4α Controls Iron Metabolism and Regulates Transferrin Receptor 2 in Mouse Liver*

**DOI:** 10.1074/jbc.M115.694414

**Published:** 2015-11-02

**Authors:** Shunsuke Matsuo, Masayuki Ogawa, Martina U. Muckenthaler, Yumiko Mizui, Shota Sasaki, Takafumi Fujimura, Masayuki Takizawa, Nagayuki Ariga, Hiroaki Ozaki, Masakiyo Sakaguchi, Frank J. Gonzalez, Yusuke Inoue

**Affiliations:** From the ‡ Division of Molecular Science, Graduate School of Science and Technology, Gunma University, Kiryu, Gunma 376-8515, Japan,; the § Department of Pediatric Oncology, Hematology and Immunology, University of Heidelberg, 69120 Heidelberg, Germany,; the ¶ Department of Cell Biology, Okayama University Graduate School of Medicine, Dentistry, and Pharmaceutical Sciences, Okayama 700-8558, Japan, and; the ‖ Laboratory of Metabolism, Center for Cancer Research, NCI, National Institutes of Health, Bethesda, Maryland 20852

**Keywords:** iron metabolism, liver, nuclear receptor, transcription regulation, transferrin, HNF4α, hepcidin, hypoferremia, transferrin receptor 2

## Abstract

Iron is an essential element in biological systems, but excess iron promotes the formation of reactive oxygen species, resulting in cellular toxicity. Several iron-related genes are highly expressed in the liver, a tissue in which hepatocyte nuclear factor 4α (HNF4α) plays a critical role in controlling gene expression. Therefore, the role of hepatic HNF4α in iron homeostasis was examined using liver-specific HNF4α-null mice (*Hnf4a*^ΔH^ mice). *Hnf4a*^ΔH^ mice exhibit hypoferremia and a significant change in hepatic gene expression. Notably, the expression of transferrin receptor 2 (*Tfr2*) mRNA was markedly decreased in *Hnf4a*^ΔH^ mice. Promoter analysis of the *Tfr2* gene showed that the basal promoter was located at a GC-rich region upstream of the transcription start site, a region that can be transactivated in an HNF4α-independent manner. HNF4α-dependent expression of *Tfr2* was mediated by a proximal promoter containing two HNF4α-binding sites located between the transcription start site and the translation start site. Both the GC-rich region of the basal promoter and the HNF4α-binding sites were required for maximal transactivation. Moreover, siRNA knockdown of HNF4α suppressed *TFR2* expression in human HCC cells. These results suggest that *Tfr2* is a novel target gene for HNF4α, and hepatic HNF4α plays a critical role in iron homeostasis.

## Introduction

Iron is an essential metallic element in living organisms and is required for the transport of oxygen by hemoglobin in red blood cells and for redox reactions by catalase and various cytochrome P450s. Because most of the iron in the human body is contained in hemoglobin and is recycled from aged red blood cells in the reticuloendothelial system, only 1–2 mg of iron are absorbed at the duodenum, and the amount exactly matches that of iron loss (1, 2). Iron deficiency causes anemia, whereas iron overload has a cytotoxic effect due to the production of reactive oxygen species (ROS).^2^ Thus, the amount of iron in the organism must be tightly regulated by various processes such as absorption, transport, cellular uptake, and storage (1–3).

Liver is the most important organ for iron metabolism, and many iron-related genes encoding transferrin (TF), transferrin receptor 2 (TFR2), and hepcidin (HAMP), for example, are highly expressed in hepatocytes. TF is a plasma iron carrier, and iron-loaded TF is mainly endocytosed into most tissues through transferrin receptor 1 (TFR1) (2–4). Congenital apotransferrinemia, deficiency of serum TF, causes iron deficiency anemia (5). Unlike TFR2, TFR1 is ubiquitously expressed in many tissues and is required for iron delivery to cells (4, 6). Mutations of the *TFR2* gene cause iron overload disease, hereditary hemochromatosis, and the affinity of TFR2 to holo-TF was 25-fold lower when compared with that of TFR1, indicating that TFR2 functions as an iron sensor unlike TFR1 (7–9). TFR2 is also an upstream regulator of HAMP, a peptide hormone secreted from liver (10). HAMP binds to iron exporter, ferroportin 1 (FPN1), and induces internalization and degradation of FPN1, resulting in decreased efflux of iron from the duodenum (11). In addition to the *TFR2* gene, mutations of the *HAMP* and *FPN1* genes also cause hereditary hemochromatosis (12). Thus, the HAMP-FPN1 axis is a central coordinator and an iron sensor in the body. Agonists and antagonists of these factors, including HAMP, are under development as therapeutic targets for iron overload disease and anemia of inflammation (1).

HNF4α is an orphan member of the nuclear receptor superfamily and positively regulates many genes involved in liver-specific functions (13, 14). HNF4α was shown to transactive at the *TF* gene through an HNF4α-binding site within the promoter region in the human hepatoma cell line (15). Moreover, an HNF4α-binding site in the *Hamp* promoter was essential for bone morphogenetic protein- and hemojuvelin-induced transactivation (16), and hepatic expression of *Hamp* mRNA was increased in liver-specific *Hnf4a*-null (*Hnf4a*^ΔH^) mice (17). These results suggest that hepatic HNF4α could be involved in the control of iron metabolism through these target genes, but the *in vivo* relationship between HNF4α and iron metabolism in adult liver has not been explored. Because *Hnf4a*^ΔH^ mice exhibit many phenotypes that attenuate liver-specific functions such as lipid and ammonia metabolism and bile acid synthesis (18–20), dysregulation of iron metabolism was also suspected in *Hnf4a*^ΔH^ mice.

In this study, *Hnf4a*^ΔH^ mice were found to exhibit iron metabolism-related phenotypes, resulting in hypoferremia, but no iron deficiency anemia and hemochromatosis was observed. Gene expression profiling revealed that hepatic expression of *Tfr2* mRNA was markedly decreased in *Hnf4a*^ΔH^ mice. Promoter analysis showed that the *Tfr2* distal promoter region containing GC-rich sequences was required for basal transactivation of the *Tfr2* gene. In addition, two HNF4α-binding sites in the proximal promoter region were essential for the maximal transactivation in combination with the proximal GC-rich sequences, revealing that *Tfr2* is a novel HNF4α target gene in liver.

## Experimental Procedures

### 

#### 

##### Animal

Liver-specific *Hnf4a*-null (*Hnf4a*^ΔH^) mice and liver-specific *Cebpa*-null (*Cebpa*^ΔH^) mice were described previously (18, 21). All experiments were performed with 45-day-old male *Hnf4a*-floxed (*Hnf4a^f/f^*) and *Hnf4a*^ΔH^ mice and 2-month-old male *Cebpa*-floxed (*Cebpa^f/f^*) and *Cebpa*^ΔH^ mice. Mice were housed in a pathogen-free animal facility under standard 12-h light/12-h dark cycle with water and chow *ad libitum*. All experiments with mice were carried out under Association for Assessment and Accreditation of Laboratory Animal Care guidelines with approval of the Animal Care and Use Committee of the NCI, National Institutes of Health, and Gunma University Animal Care and Experimentation Committee.

##### Serum and Liver Iron Measurements

Mice were anesthetized with 2.5% avertin and decapitated, and trunk blood was collected for the measurement of red blood cells (RBC), hemoglobin, hematocrit, and the mean cell volume, or collected in a serum separator tube (BD Biosciences). The serum was separated by centrifugation at 7,000 × *g* for 5 min and stored at −20 °C prior to analysis. The serum iron concentration and the unsaturated iron-binding capacity were determined colorimetrically using iron and the total iron-binding capacity kit (Sigma). The total iron-binding capacity was calculated by adding the serum iron concentration and the unsaturated iron-binding capacity. Transferrin saturation (%) was calculated as (serum iron concentration/total iron-binding capacity) × 100. Livers were perfused with phosphate-buffered saline (PBS) to remove serum iron, excised, and dried at 100 °C for 16 h. Dried liver samples were dissolved in acidic solution (3 n HCl, 0.6 m trichloroacetic acid) and incubated at 65 °C for 20 h. Extracted iron solution was reacted with chromogen solution (1.25 m acetic acid (pH 4.7), 0.008% bathophenanthroline, 0.08% glyceric acid) at room temperature for 30 min, and the liver iron was determined colorimetrically at 510 nm.

##### IronChip Analysis

For the cDNA microarray experiments, the “IronChip” (version 3.0) was used. Experimental details concerning the selection of the cDNA clones, the preparation of the microarray platform, the synthesis of fluorescent cDNA probes, prehybridization, and hybridization conditions of the microarrays as well as scanning and data analysis are described elsewhere (22–24). The symbols of the genes mentioned and RefSeq accession number in the text are summarized in Table 4.

##### RNA Extraction, Reverse Transcription, and Real Time PCR

Total RNA from the mouse livers and HepG2 cells was extracted using Tripure Isolation Reagent (Roche Applied Science, Tokyo, Japan). cDNA was transcribed using ReverTra Ace qPCR RT Master Mix with gDNA Remover (TOYOBO, Osaka, Japan), and real time PCR was performed using FastStart SYBR Green Master Mix (Roche Applied Science) with the specific primers on LightCycler 480 System II (Roche Applied Science). Levels of mRNA expression were normalized relative to *Gapdh* mRNA as an internal control using the ΔΔ*Ct* method. The following primers were used for real time PCR: mouse *Hnf4a* (agaggttctgtcccagcagatc and cgtctgtgatgttggcaatc); human *HNF4A* (caggctcaagaaatgcttcc and ggctgctgtcctcatagctt); mouse *Tfr2* (ctatctggtcctgatcaccct and tcagggttgacatcttcatcga); human *TFR2* (gtggaccgacacgcactac and tgtaggggcagtagacgtcag); mouse *Gapdh* (gacttcaacagcaactcccac and tccaccaccctgttgctgta); human *GAPDH* (agccacatcgctcagacac and gcccaatacgaccaaatcc); mouse *Tf* (cgcagtcctcttgagaaagc and agcctgggcacagttgac); mouse *Hjv* (gccaacgctaccaccatc and tcaaaggctgcaggaagatt); mouse *Ftl* (ttttgatcgggatgacgtg and cgttctgaaactcgaggagac); mouse *Fth* (tggagttgtatgcctcctacg and tggagaaagtatttggcaaagtt); mouse *Hpx* (ggaagaatcccatcacctca and caggagggtacacccagact); mouse *Cat* (ccttcaagttggttaatgcaga and caagtttttgatgccctggt); mouse *Sod1* (ccatcagtatggggacaataca and ggtctccaacatgcctctct); mouse *Gpx1* (ggtttcccgtgcaatcagt and tcggacgtacttgagggaat); mouse *Tfr1* (tggaatcccagcagtttctt and gctgctgtacgaaccatttg); mouse *Hamp* (gatggcactcagcactcg and ctgcagctctgtagtctgtctca); mouse *Hfe* (ggaaaaggaaggcttcagga and cctccaagtctttggctgag); mouse *Fpn1* (tggccactctctctccactt and tgtcaagagaaggctgtttcc); mouse *Heph* (tctatacatgcccatggagttct and tgggatgttccactggtaagt); mouse *Cp* (gggccaatgaaaatatgcaa and tcaaacactgtgggaaacaagt); and mouse *Cebpa* (tggacaagaacagcaacgag and tcactggtcaactccagcac).

##### Cloning of Promoter Region of Mouse Tfr2 Gene

The −1972, −982, −396, −241, −116, −82, −66, −46/+67, −116, −46, +505/+1144, −46, −116/+504, and −116/+896 fragments from the transcription start site of the mouse *Tfr2* promoter containing KpnI and XhoI sites were amplified with genomic DNA from mouse liver by PCR and cloned into the luciferase reporter vector, pGL4.11 (Promega, Madison, WI). HSV-TK mini and HSV-TK promoters were amplified with pGL4.74 (Promega) by PCR and cloned into EcoRV and BglII/HindIII sites of the pGL4.11 and named pGL4.11/TK and pGL4.11/TK mini, respectively. Mutations were introduced into GC-rich sequences and HNF4α-binding sites in the *Tfr2* promoter by overlapped or inverted PCR-based site-directed mutagenesis. The following primers were used for amplification of HSV-TK mini promoter (ggcatagatctttcgcatattaaggtgacgcgtgtggcctc and ggcataagcttttaagcgggtcgctgcagggtcgctcggtg) and the TK promoter (gcataagcttaaatgagtcttcggacctcg and ggcataagcttttaagcgggtcgctgcagggtcgctcggtg). Similarly, the following primers were also used for PCR-based mutagenesis of GC-1 region (**atttgtt**agtcctctggggcgg and **aacaaat**cgcacgtccttttct), GC-2 region (**tttacatttg**ctgggggcgtgctct and **caaatgtaaa**agaggactccgcccc), GC-3 region (**taaactaa**ctctagtgggtgtgg and **ttagttta**caggccccgccccag), the HNF4α-binding site at +182/+201 (**t**gac**g**ctg**tga**tttcccattcacagctgc and acctcacccttacttctggtccagg), and the distal HNF4α-binding site at +830/+842 (**cgc**gtctgctctgaggtttaaaaaa and ggccaacttggttcccgtcacaggt). The induced mutations are indicated as bold and underlined.

##### Transient Transfection and Luciferase Assays

HepG2 and HEK293T cells were cultured at 37 °C in Dulbecco's modified Eagle's medium (WAKO, Osaka, Japan) containing 10% fetal bovine serum (HyClone, Logan, UT) and 100 units/ml penicillin/streptomycin (Invitrogen). For suspension transfection, wild type or mutated *Tfr2* promoters cloned into pGL4.11 and pGL4.74 encoding *Renilla* luciferase regulated by the HSV-TK promoter as an internal control were transfected into HepG2 cells with polyethyleneimine max (Polyscience, Warrington, PA) as a transfection reagent. For co-transfection using HEK293T cells, HNF4α expression plasmid, pSG5/HNF4α, was used (19). After 48 h, the cells were washed with phosphate-buffered saline (PBS), and promoter activities were measured using Dual-Glo Luciferase Assay System (Promega).

##### Transfection of siRNA

Two kinds of siRNA for human HNF4α (10 nm, Sigma, Tokyo, Japan) were independently transfected into HepG2 cells with Lipofectamine RNAiMAX (Life Technologies, Inc.). After 48 h, cells were trypsinized and re-transfected with 10 nm of the same siRNA. After 48 h of re-transfection, cells were harvested, and total RNA was extracted using Tripure Isolation Reagent for RT quantitative PCR. Nucleotide sequences for the siRNA duplexes targeting human HNF4α are follows: GGCAGUGCGUGGUGGACAA for siHNF4α-1 and AGAGAUCCAUGGUGUUCAA for siHNF4α-2.

##### Western Blot

Liver samples from *Hnf4a^f/f^* and *Hnf4a*^ΔH^ mice were homogenized in lysis buffer (7 m urea, 2 m thiourea, 1% Triton X-100) and allowed to sit on ice for 30 min. The homogenate was centrifuged at 12,000 × *g* for 30 min at 4 °C, and the supernatants were used as whole cell lysates. The whole cell lysates and serum protein (40 μg), determined by Quick Start^TM^ Bradford Dye Reagent (Bio-Rad), were diluted with Laemmli sample buffer, incubated at 65 °C for 15 min, and fractionated by 10% SDS-PAGE. The gels were stained with Coomassie Brilliant Blue R-250 or transferred onto a PVDF membrane (GE Healthcare, Tokyo, Japan). The membrane was incubated for 1 h with PBS containing 0.1% Tween 20 and 5% skim milk and then incubated for 1 h with anti-transferrin (Santa Cruz Biotechnology, Dallas, TX), anti-transferrin receptor 2 (Abcam, Tokyo, Japan), and anti-γ-tubulin (TUBG) (Sigma) antibodies. After washing, the membrane was incubated for 1 h with horseradish peroxidase-conjugated secondary antibodies (Santa Cruz Biotechnology), and the reaction product was visualized using SuperSignal West Pico Chemiluminescent Substrate (Pierce). Expression of TF and TFR2 proteins was quantified by densitometric analysis using ImageJ software and the expression in *Hnf4a*^ΔH^ was presented as expression differences relative to the *Hnf4a*^f^*^f/f^* mice.

##### Gel Mobility Shift Analysis

Nuclear extracts from HepG2 cells were prepared using NE-PER nuclear and cytoplasmic extraction reagents (Thermo Fisher Scientific, Yokohama, Japan), and gel shift analysis was carried out using LightShift Chemiluminescent EMSA kit (Thermo Fisher Scientific). The following double-stranded probes were used (mutations are indicated as bold and underlined); the HNF4α-binding site at +182/+201 in the mouse *Tfr2* promoter (wild type, gggtgaggtggaccctgaactttcccattcacagctgca and tgcagctgtgaatgggaaagttcagggtccacctcaccc; mutant, gggtgaggt**t**gac*g*ctg**tga**tttcccattcacagctgca and tgcagctgtgaatgggaaa**tca**cag***c***gtc**a**acctcaccc); the HNF4α-binding site at +830/+842 in the mouse *Tfr2* promoter (wild type, gaaccaagttggccaaagtctgctctgaggt and acctcagagcagactttggccaacttggttc; mutant, gaaccaagttggcc**cgc**gtctgctctgaggt and acctcagagcagac**gcg**ggccaacttggttc); and the HNF4α-binding site at −203/−192 in the mouse ornithine transcarbamylase (*Otc*) promoter as a positive control (gttaggcttaaagttcaagtg and cacttgaactttaagcctaac) (19). Nuclear extracts (3 μg) and the 5′-biotin-labeled probes of the HNF4α-binding sites for the *Tfr2* promoter (wild type) were added, and the reaction mixture was incubated on ice for 10 min. For competition experiments, a 50-fold excess of unlabeled probe was added to the reaction mixture, and the mixture was incubated on ice for 10 min prior to the addition of the 5′-biotin-labeled probe. For supershift analysis, 1 μg of anti-HNF4α or anti-peroxisome proliferator-activated receptor α antibodies (Santa Cruz Biotechnology) was added to the reaction mixture, and the mixture was incubated on ice for 10 min after the addition of the 5′-biotin-labeled probe. DNA-protein complexes were fractionated by 7% PAGE containing 5% glycerol and blotted onto a Biodyne B nylon membrane (Pall, Tokyo, Japan). After washing, DNA-protein complexes were visualized using a detection module in the kit on an ImageQuant LAS4000.

##### Chromatin Immunoprecipitation

HepG2 cells cultured in a 10-cm dish were fixed in 0.5% formaldehyde for 10 min and then quenched with 125 mm glycine for 5 min at room temperature. After washing with ice-cold PBS, the cells were resuspended in 3 ml of Lysis buffer 1 (50 mm HEPES-KOH (pH 7.5), 140 mm NaCl, 1 mm EDTA, 10% glycerol, 0.5% Nonidet P-40, 0.25% Triton X-100, and protease inhibitor (Roche Applied Science)) on ice for 10 min and then centrifuged at 1,400 × *g* for 5 min. The cell pellet was resuspended in 3 ml of Lysis buffer 2 (10 mm Tris-HCl (pH 8.0), 200 mm NaCl, 1 mm EDTA, and 5 mm EGTA) for 10 min at room temperature and then centrifuged at 1,400 × *g* for 5 min. The cell pellet was resuspended in 1 ml of Lysis buffer 3 (10 mm Tris-HCl (pH 8.0), 300 mm NaCl, 1 mm EDTA, 0.5 mm EGTA, and 0.1% sodium deoxycholate). Liver samples stored at −80 °C were ground to pieces with a pestle and mortar under liquid nitrogen and fixed in PBS containing 20 mm sodium butyrate, 1% formaldehyde, and protease inhibitor mixture for 10 min at room temperature. After centrifugation, the pellet was resuspended in Lysis buffer (50 mm Tris-HCl (pH 8.0), 10 mm EDTA, 1% SDS, and 20 mm sodium butyrate). The cell lysate from HepG2 cells and liver samples was disrupted by an ultrasonicator (UR-20P, TOMY, Tokyo, Japan) for 5 min on ice and then 1% Triton X-100 was added, followed by centrifuging at 20,000 × *g* for 10 min. A small volume of the supernatant was stored at 4 °C as the input samples. The remaining supernatant was pre-cleared by adding of 15 μl of a 50% slurry of protein G-Sepharose 4 Fast Flow (GE Healthcare) with sonicated salmon sperm DNA and rotated for 4 h at 4 °C, followed by centrifuging at 1,900 × *g* for 5 min. The supernatant was divided into two pieces and incubated with anti-HNF4α antibody (4 μg, Santa Cruz Biotechnology) or control normal goat IgG for 16 h at 4 °C. The reaction mixture was centrifuged at 1,900 × *g* for 5 min at 4 °C, and the pellet collected was washed for 5 min at 4 °C with 1 ml of RIPA-1 buffer (50 mm Tris-HCl (pH 8.0), 150 mm NaCl, 1 mm EDTA, 1% Triton X-100, 0.1% SDS, and 0.1% sodium deoxycholate), RIPA-2 buffer (50 mm Tris-HCl (pH 8.0), 300 mm NaCl, 1 mm EDTA, 1% Triton X-100, 0.1% SDS, and 0.1% sodium deoxycholate), LiCl wash solution (10 mm Tris-HCl (pH 8.0), 0.25 m LiCl, 1 mm EDTA, 0.5% Nonidet P-40, and 0.5% sodium deoxycholate), and TE (10 mm Tris-HCl (pH 8.0) and 1 mm EDTA), respectively. Then the ChIP direct elution buffer (10 mm Tris-HCl (pH 8.0), 300 mm NaCl, 5 mm EDTA, and 0.5% SDS) was added to the pellet and incubated for 16 h at 65 °C for decross-linking. After treatment with RNase A for 30 min at 37 °C and proteinase K for 2 h at 55 °C, the DNA was purified and used for PCR and real time PCR. Enrichment of the HNF4α binding was normalized to the input samples using the ΔΔ*Ct* method and expressed as fold-enrichment as compared with the control normal IgG antibody. The following primers were used for amplification of the HNF4α-binding sites in the human *TFR2* promoter (gggaactaggaggccaaagt and tctcccctgccaatctctc), the human *MIR-194/192* gene without HNF4α-binding site as a negative control (ccttgtgagggcacaccttt and aaagccaggcagtcagtgct), the HNF4α-binding sites in the mouse *Tfr2* promoter (caggaagaccggctaacg and tcttgggtctggttgctagg), and the mouse *Hmgcs2* gene without HNF4α-binding site as a negative control (gatccctgggactcacaca and gaatgcacatttatggaggtca).

##### Statistical Analysis

All values are expressed as the mean ± S.D. All data were analyzed by the unpaired Student's *t* test for significant differences between the mean values of each group.

## Results

### 

#### 

##### Hnf4a^ΔH^ Mice Exhibit Hypoferremia

Hematological parameters are good indicators for alternation of iron homeostasis. Here, hepatic HNF4α was found to maintain serum iron levels (Table 1). *Hnf4a*^ΔH^ mice exhibited a 60% reduction in serum iron compared with control *Hnf4a^f/f^* mice. Likewise, total iron binding capacity, an index of the total amount of serum iron that transferrin can bind, was also reduced in *Hnf4a*^ΔH^ mice, indicating a lower availability of transferrin protein in *Hnf4a*^ΔH^ mice. However, because no significant difference in transferrin saturation was observed in *Hnf4a*^ΔH^ mice, iron-binding transferrin is normal in *Hnf4a*^ΔH^ mice. Moreover, red blood cell counts (RBC), hemoglobin levels, the hematocrit, and the mean cell volume were unchanged in *Hnf4a*^ΔH^ mice compared with *Hnf4a*^ΔH^ mice (Table 2). Furthermore, no significant difference in liver iron content was detected in *Hnf4a*^ΔH^ mice (Table 3), suggesting that *Hnf4a*^ΔH^ mice do not develop liver iron deficiency disease nor hereditary hemochromatosis.

**TABLE 1:** **Serum iron content in *Hnf4a*^ΔH^ mice** Values are presented as mean ± S.D. (*n* = 8). TIBC is total iron-binding capacity. Significant differences are compared with *Hnf4a^f/f^* mice.

Mice	Iron	TIBC	Transferrin saturation
	μ*g/dl*	μ*g/dl*	%
*Hnf4a^f/f^*	235.7 ± 43.1	751.8 ± 223.1	34.6 ± 15.3
*Hnf4a*^ΔH^	95.7 ± 22.8*^a^*	336.9 ± 150.9*^a^*	34.8 ± 19.8

*^a^ p* < 0.005.

**TABLE 2:** **Hematological parameters in *Hnf4a*^ΔH^ mice** RBC is red blood cells; HGB is hemoglobin; MCV is mean corpuscular volume; HCT is hematocrit. All values are presented as means ± S.D. (*n* = 3–5).

Mice	RBC	HGB	MCV	HCT
	*m*/μ*l*	*g/dl*	*fl*	%
*Hnf4a^f/f^*	9.2 ± 0.1	14.3 ± 0.1	49.6 ± 2.0	45.7 ± 1.5
*Hnf4a*^ΔH^	10.2 ± 0.2	14.8 ± 1.3	47.6 ± 2.8	49.1 ± 3.6

**TABLE 3:** **Liver iron content in *Hnf4a*^ΔH^ mice** Values are presented as mean ± S.D. (*n* = 5).

Mice	Iron
	μ*g/g liver*
*Hnf4a^f/f^*	235 ± 92
*Hnf4a*^ΔH^	200 ± 108

##### Expression of Genes Involved in Iron Metabolism is Altered in Hnf4a^ΔH^ Mice

**FIGURE 1.**
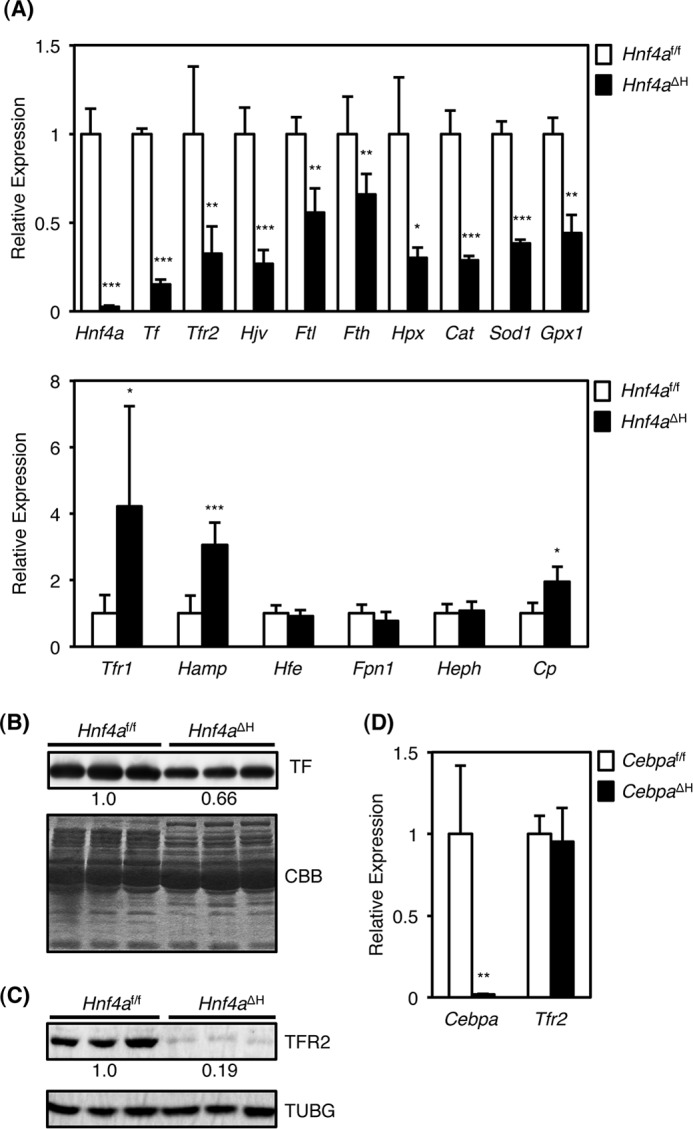
**Hepatic expression of iron metabolism-related genes in *Hnf4a*^ΔH^ mice.** *A,* real time PCR for *Hnf4a*, *Tf*, *Tfr2*, *Hjv*, *Ftl*, *Fth*, *Hpx*, *Cat*, *Sod1*, and *Gpx1* (*upper panel*), and *Tfr1*, *Hamp*, *Hfe*, *Fpn1*, *Heph*, and *Cp* (*lower panel*) mRNAs from total liver RNA of *Hnf4a*^ΔH^ and *Hnf4a^f/f^* mice (*n* = 5 for each genotype). The normalized expression in *Hnf4a*^ΔH^ mice was presented relative to that in *Hnf4a^f/f^* mice. *B,* Western blot of TF protein from serum of *Hnf4a*^ΔH^ and *Hnf4a^f/f^* mice (*n* = 3 for each genotype) (*upper panel*). Coomassie Brilliant Blue stain of serum total protein of *Hnf4a*^ΔH^ and *Hnf4a^f/f^* mice as loading controls (*lower panel*) is shown. The expression of TF protein was quantified by densitometric analysis using ImageJ software. The normalized TF expression in *Hnf4a*^ΔH^ was presented as relative to that in *Hnf4a^f/f^* mice. *C,* Western blot of TFR2 (*upper panel*) and TUBG (*lower panel*) protein from livers of *Hnf4a*^ΔH^ and *Hnf4a^f/f^* mice (*n* = 3 for each genotype). The TFR2 expression normalized by TUBG in *Hnf4a*^ΔH^ was presented as relative to *Hnf4a^f/f^* mice. *D,* real time PCR for *Cebpa a*nd *Tfr2* mRNAs from total liver RNA of *Cebpa*^ΔH^ and *Cebpa^f/f^* mice (*n* = 5 for each genotype). The normalized expression in *Cebpa*^ΔH^ mice was presented relative to that in *Cebpa^f/f^* mice. Data are mean ± S.D. *, *p* < 0.05; **, *p* < 0.01; ***, *p* < 0.001.

The expression profile of mRNAs encoded by iron metabolism-related genes was analyzed in *Hnf4a*^ΔH^ and *Hnf4a^f/f^* mouse livers using IronChip microarray analysis (Table 4). Hepatic expression of many mRNAs significantly changed in *Hnf4a*^ΔH^ mice. Among the down-regulated mRNAs were transferrin (*Tf*), transferrin receptor 2 (*Tfr2*), hemojuvelin (*Hjv*), ferritin light chain (*Ftl*), ferritin heavy chain (*Fth*), hemopexin (*Hpx*), catalase (*Cat*), Cu-Zn superoxide dismutase (*Sod1*), and glutathione peroxidase 1 (*Gpx1*). Up-regulated mRNAs included transferrin receptor 1 (*Tfr1*), hepcidin (*Hamp*), and ceruloplasmin (*Cp*) mRNAs. Microarray data were validated by quantitative RT-PCR (Fig. 1*A*). Because atransferrinemia/hypotransferrinemia is caused by recessive mutations in the *Tf* gene resulting in iron-deficient hypochromic anemia in humans and mice (25, 26), reduced expression of TF may cause hypoferremia. Indeed, as expected from reduced *Tf* mRNA expression, serum TF protein in *Hnf4a*^ΔH^ mice was decreased by 66% compared with *Hnf4a^f/f^* mice (Fig. 1*B*). These data show that misregulation of a previously validated HNF4α target gene is preserved in *Hnf4a*^ΔH^ mice (15). Thus, HNF4α-dependent regulation of *Tf* could partially contribute to the hypoferremia observed in this mouse model.

**TABLE 4:** **Expression profiling of hepatic mRNA involved in iron metabolism in *Hnf4a*^ΔH^ mice** Microarray analysis was performed using pooled total RNA from liver tissues of *Hnf4a*^ΔH^ and *Hnf4a^f/f^* mice. Ratio of expression levels between both of them was indicated as fold-change.

Gene symbol	Ref. Seq. (NM_)	Fold-change (*Hnf4a*^ΔH^/*Hnf4a^f/f^*)	Gene symbol	Ref. Seq. (NM_)	Fold-change (*Hnf4a*^ΔH^/*Hnf4a^f/f^*)
*Tf*	133977	0.17	*Irp1*	007386	0.72
*Ltf*	008522	0.19	*Fth*	010239	0.73
*Mgst1*	019946	0.29	*Zfp36*	011756	0.82
*Tfr2*	015799	0.30	*Fmo2*	018881	1.31
*Atp7b*	007511	0.33	*Npm1*	008722	1.38
*Selp*	011347	0.33	*Hif1a*	010431	1.39
*Cat*	009804	0.38	*Fmo1*	010231	1.42
*Efna1*	010107	0.44	*Mt3*	013603	1.47
*Gpx1*	008160	0.48	*Srsf3*	013663	1.52
*Sod1*	011434	0.48	*Por*	008898	1.53
*Ldh1*	010699	0.50	*Cox17*	001017429	1.55
*Rplp0*	007475	0.51	*Arg1*	007482	1.58
*Atp7a*	009726	0.52	*Maob*	172778	1.61
*Prodh*	011172	0.53	*Hamp*	032541	1.68
*Nr1i3*	009803	0.56	*Hspa8*	031165	1.71
*Ftl*	010240	0.58	*Cp*	007752	1.72
*Acss2*	019811	0.58	*Hspa5*	022310	1.72
*Slc31a1*	175090	0.60	*Psap*	011179	1.72
*Hpx*	017371	0.63	*App*	007471	1.88
*Acox3*	030721	0.63	*Gsn*	146120	1.89
*Atox1*	009720	0.64	*Tfr1*	011638	3.31
*Impa1*	018864	0.64	*Mt1*	013602	3.80
*Cyp3a13*	007819	0.64	*Fmo3*	008030	4.34
*Cyp1a1*	009992	0.65	*Spp1*	009263	5.05
*Txn1*	011660	0.70	*Mt2*	008630	6.19
*Nfs1*	010911	0.70			

In addition, decreased expression of hepatic *Tfr2* mRNA to 30% was observed in *Hnf4a*^ΔH^ mice, although expression of *Tfr1* mRNA encoding a receptor with affinity to holo-TF 25-fold higher than TFR2 was increased to 4-fold (Fig. 1*A*) (8). TFR2 protein in *Hnf4a*^ΔH^ mice was also decreased by 19% compared with *Hnf4a^f/f^* mice (Fig. 1*C*). Because TFR2 and HNF4α are highly expressed in the liver and mutations in the *TFR2* gene cause type III hemochromatosis in humans (6, 7), further studies were focused on the *Tfr2* gene. Previous reports suggest that the promoter activity of the *Tfr2* gene was induced by C/EBPα expression (27). However, expression of hepatic *Tfr2* was unchanged in liver-specific *Cebpa*-null (*Cebpa*^ΔH^) mice (Fig. 1*D*), indicating that C/EBPα is unlikely to be the main regulator of *Tfr2* expression in the liver.

##### Promoter Analysis of the Distal Region of the Tfr2 Promoter

**FIGURE 2.**
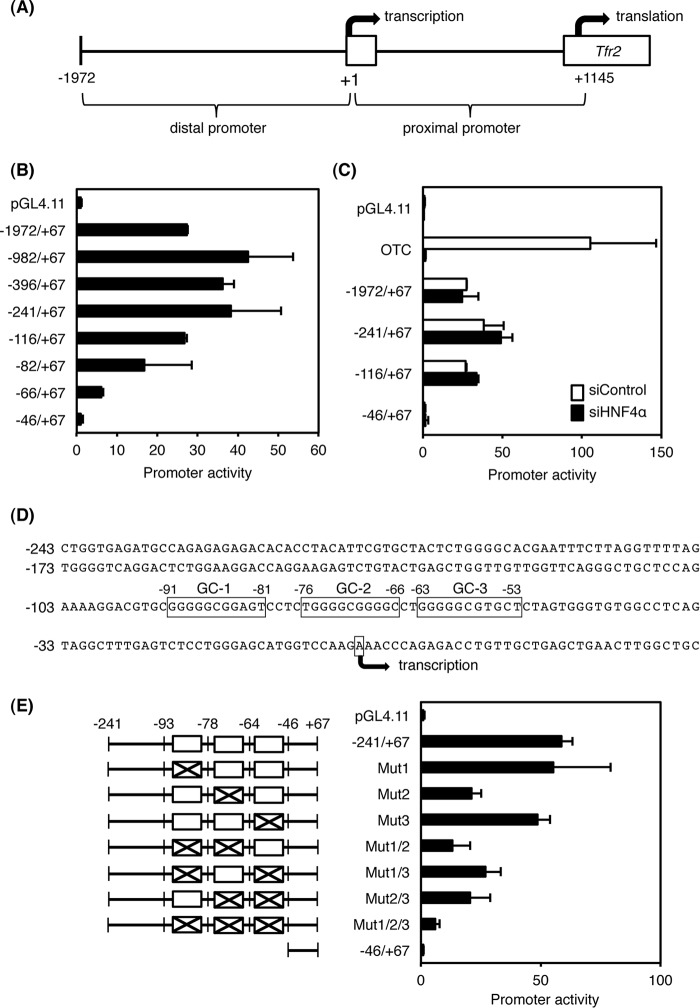
**Promoter analysis of distal region of the mouse *Tfr2* gene.** *A,* schematic structure of the promoter region of the mouse *Tfr2* gene. *B,* promoter activity of the distal region of the *Tfr2* gene in HepG2 cells. *C,* promoter constructs were transfected into HepG2 cells with 20 nm siRNA for HNF4α (*siHNF4*α) or negative control (*siControl*). *Otc* promoter whose transactivation is dependent on HNF4α expression was used as a positive control. *D,* nucleotide sequences of the distal promoter of the *Tfr2* gene. Three GC-rich sequences (GC-1, -2, and -3) are *boxed. E,* mutations were introduced into three GC-rich sequences (1–3) between −93 and −46 in the −241/+67 promoter (*left panel*). The promoter activities were measured in HepG2 cells (*right panel*). The normalized activity ± S.D. (*n* = 3) was presented as promoter activity based on pGL4.11.

Promoter analysis of the mouse *Tfr2* gene was performed to determine whether HNF4α directly regulates *Tfr2* expression. Analysis of the distal promoter was carried out in HepG2 cells that highly express endogenous HNF4α and TFR2 (6) revealing significant promoter activities with the region between −116 and −46 from the transcription start site (Fig. 2*B*). Activity of the *Otc* promoter that has two HNF4α-binding sites was completely suppressed by HNF4α knockdown using siRNA; however, no repression of the promoter activities was detected in all *Tfr2* promoters by siRNA against HNF4α (Fig. 2*C*), indicating that the distal promoter of the *Tfr2* gene was activated in an HNF4α-independent manner. Searching the JASPER database for transcription factor-binding sites in the region between −116 and −46 revealed three GC boxes (GC-1, 2, and -3) that were predicted between −91 and −53 (Fig. 2*D*). Furthermore, the stepwise transactivation of the promoter activity was observed in the presence of these GC boxes (Fig. 2*B*). To determine which regions of these GC boxes are sufficient for transactivation, mutations were introduced into each GC box. Mutations of the GC-2 region significantly suppressed the promoter activity, and mutations of all GC regions further suppressed the activity (Fig. 2*E*), indicating that the GC-2 region is a central *cis*-element for transactivation, and the GC-1 and GC-3 regions are required for the maximal transactivation of the distal promoter.

##### Promoter Analysis of the Proximal Region of the Tfr2 Promoter

**FIGURE 3.**
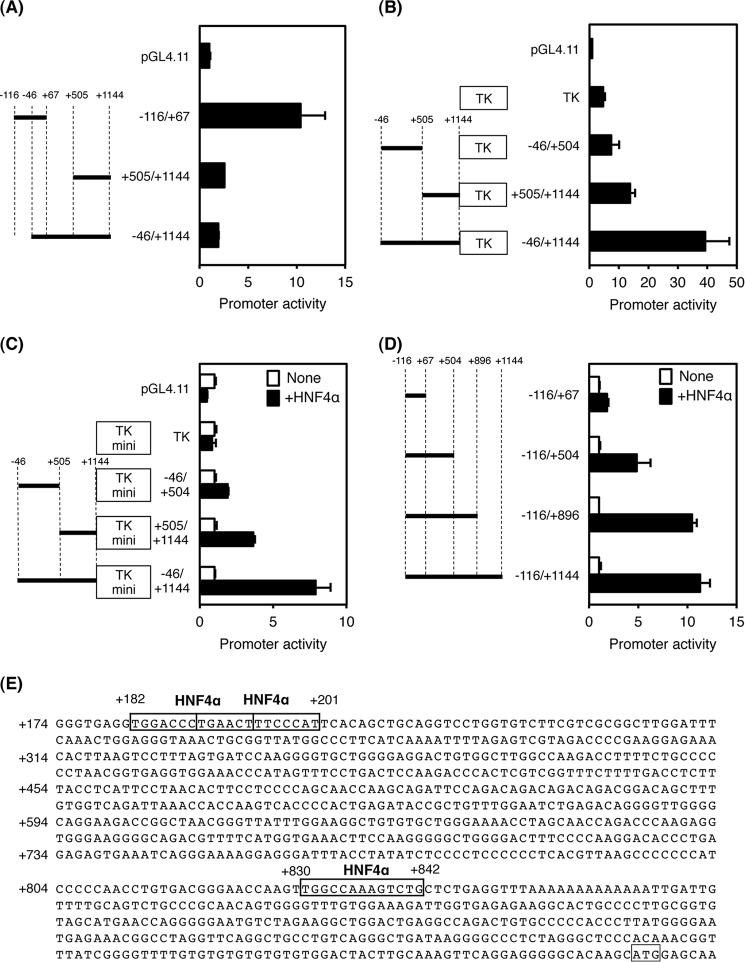
**Promoter and enhancer analysis of the proximal region of the mouse *Tfr2* gene.** *A,* promoter activity of the proximal region of the *Tfr2* gene in HepG2 cells. *B,* enhancer activity of the proximal region of the *Tfr2* gene with TK promoter. *C,* enhancer activity of the proximal region of the *Tfr2* gene with TK mini promoter in HEK293T cells. Empty vector (*None*), or HNF4α expression vector (+*HNF4*α) was co-transfected. *D,* enhancer activity of the proximal region of the *Tfr2* gene with the distal promoter in HEK293T cells. Empty vector or HNF4α expression vector was also co-transfected. *E,* nucleotide sequences of the proximal promoter of the *Tfr2* gene. Predicted HNF4α-binding sites are *boxed*. The normalized activity ± S.D. (*n* = 3) is presented as promoter activity based on pGL4.11 (*A* and *B*) or empty vector-transfected control (*C* and *D*).

**FIGURE 4.**
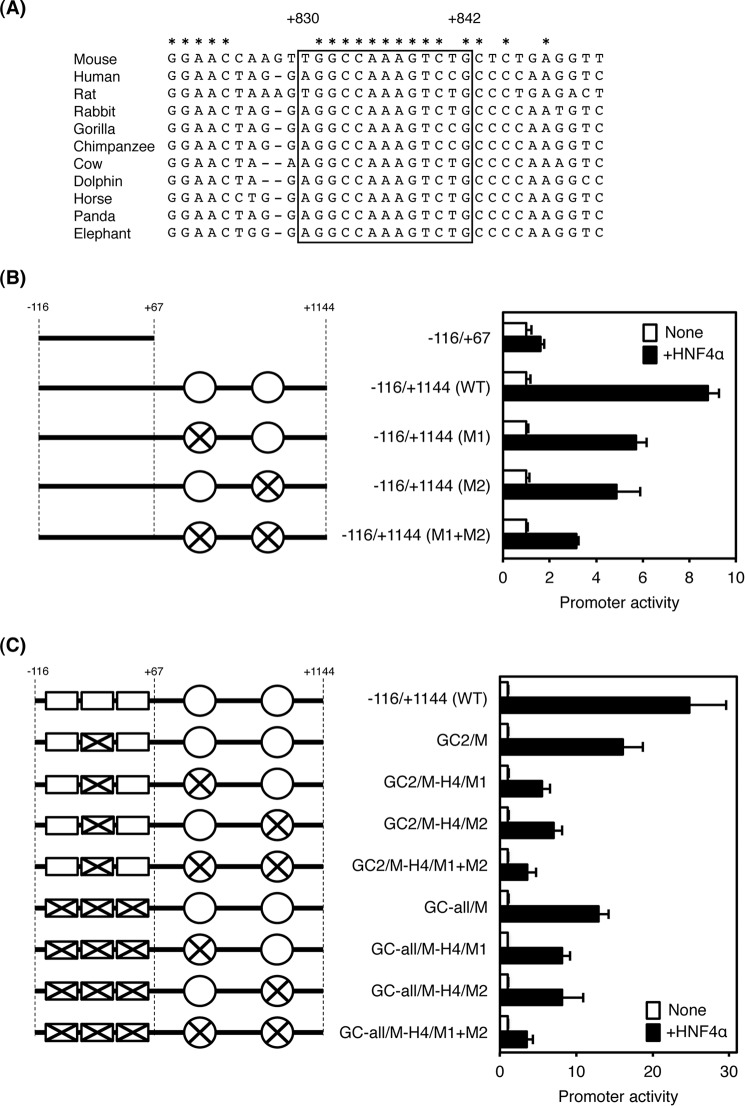
**Requirement of the minimal promoter of the *Tfr2* gene for transactivation by HNF4α.** *A,* sequence alignment of the proximal promoter of the *Tfr2* gene in mouse, human, rat, rabbit, gorilla, chimpanzee, cow, dolphin, horse, panda, and elephant. Predicted HNF4α-binding site is *boxed*. Completely conserved nucleotides among the species are shown by *asterisks. B,* mutations were introduced into the HNF4α-binding sites in the proximal promoter of the *Tfr2* gene. Empty vector or HNF4α expression vector was co-transfected with the mutated proximal promoter and the minimal distal promoter, and the enhancer activity was measured. *C,* mutations were introduced into the HNF4α-binding sites and GC-rich sequences in the *Tfr2* promoter. Empty vector or HNF4α expression vector was co-transfected with the mutated promoter, and the enhancer activity was measured. The normalized activity ± S.D. (*n* = 3) is presented as promoter activity based on empty vector-transfected control.

As described above, regions that respond to HNF4α were not identified in the distal promoter. No transactivation was detected in this promoter region (+505/+1144 and −46/+1144) as compared with the promoterless and the distal promoter (−116/+67, see Fig. 3*A*). Therefore, the proximal promoter was analyzed for the presence of an HNF4α-dependent enhancer. At first, the full-length proximal promoter (−46/+1144) had enhancer activity in the presence of the core *Tk* promoter (Fig. 3*B*). In addition, the shorter promoter regions (−46/+505 and −46/+1144) had weak enhancer activities, showing that these regions might cooperatively contribute to enhancer transactivation. Next, the −46/+504 and +505/+1144 proximal promoters were transactivated by HNF4α expression vector, and the full-length promoter was further transactivated by HNF4α in the presence of the *Tk* mini promoter (Fig. 3*C*). These results indicate that the proximal promoter contains at least two HNF4α-dependent elements located at −46/+504 and +505/+1144. In addition, the proximal promoters with the distal promoter (−116/+504, −116/+896, and −116/+1144) was also transactivated by HNF4α as compared with the distal promoter alone (−116/+67), with the result that HNF4α has the potential to transactivate the native *Tfr2* promoter via two regions located at +67/+504 and +505/+896 (Fig. 3*D*). By searching with the JASPER database, three potential HNF4α-binding sites were found at +182/+194, +189/+201, and +830/+842, and the binding sites located at +182/+194 and +189/+201 overlapped (Fig. 3*E*). Of these, the binding site at +830/+842 was highly conserved among species (Fig. 4*A*). To determine whether these predicted HNF4α-binding sites could transactivate the *Tfr2* gene in an HNF4α-dependent manner, mutations were introduced into the binding sites. Because the binding site at +182/+194 and +189/+201 overlapped, mutations were introduced into the middle region at +182/+201 so that HNF4α could not bind to both sites. Consequently, disruption of each binding site decreased HNF4α-dependent transactivation, and disruption of both binding sites further repressed the transactivation, indicating that these HNF4α-binding sites were transactivated in an HNF4α-dependent manner (Fig. 4*B*).

To investigate whether the minimal promoter region in the distal promoter would be essential for transactivation by HNF4α, mutations were introduced into both the GC boxes and HNF4α-binding sites (Fig. 4*C*). In the presence of the HNF4α-binding sites in the proximal promoter, mutations of the GC-2 region (GC2/M) inhibited the transactivation by HNF4α by about 35% as compared with the wild-type promoter (−116/+1144WT). Mutations of each HNF4α-binding site with mutations of the GC-2 region (GC2/M-H4/M1 and GC2/M-H4/M2) markedly suppressed the transactivation by HNF4α, and further suppression was detected by mutations in both HNF4α-binding sites (GC2/M-H4/M1+M2). Additionally, mutations of all GC boxes (GC-all/M) further inhibited the transactivation by HNF4α when compared with that of the mutations of the GC-2 region alone (GC2/M). Transactivation by HNF4α was particularly inhibited by mutations in all GC regions and HNF4α-binding sites, indicating that the GC-2 region plays a critical role in transactivation of the *Tfr2* gene by HNF4α.

##### Direct Binding of HNF4α to the Proximal Region of the Tfr2 Promoter

**FIGURE 5.**
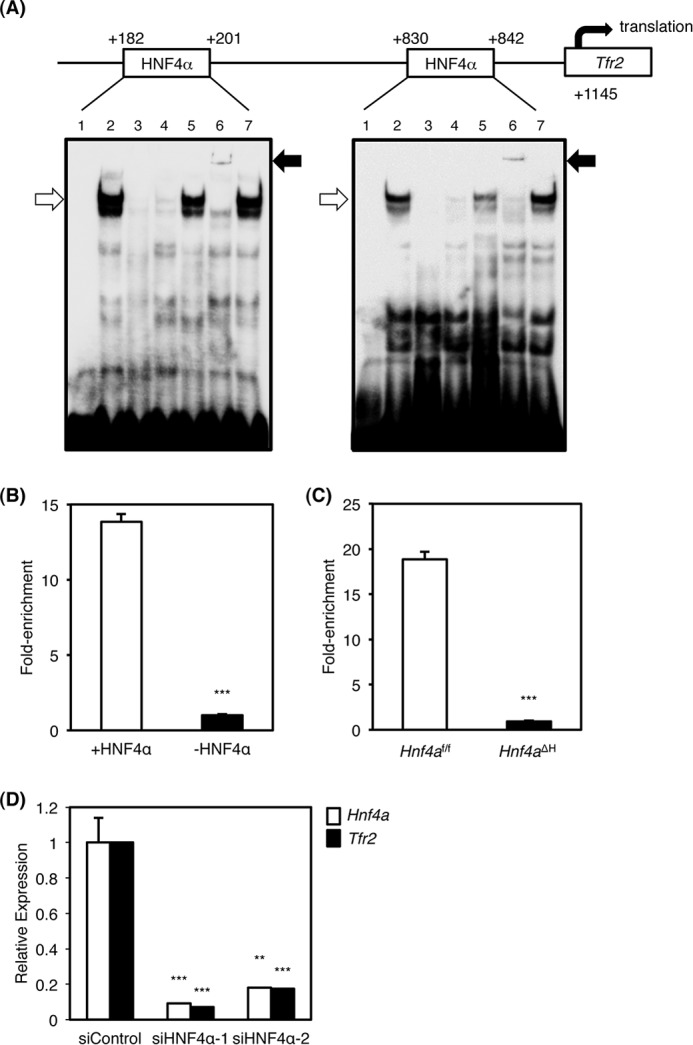
**Identification of HNF4α-binding sites in the *Tfr2* promoter and HNF4α-dependent transactivation of the *Tfr2* gene.** *A,* gel mobility shift analysis. Nuclear extracts from HepG2 cells were incubated with the probe carrying the distal (*left panel*) and proximal (*right panel*) HNF4α-binding sites in the *Tfr2* promoter in the absence (*lane 2*) or presence of the unlabeled *Tfr2*, the *Otc*, and the mutated *Tfr2* probes (*lanes 3–5*). For supershift analysis, anti-HNF4α and anti-PPARα antibodies were added (*lanes 6* and *7*). *B,* chromatin immunoprecipitation using HepG2 cells were performed with anti-HNF4α antibody and normal goat IgG. The regions with the proximal HNF4α-binding sites (+*HNF4*α) and the regions without HNF4α-binding site (−*HNF4*α) in the human *MIR-194/192* gene were amplified. The data from real time PCR were normalized relative to the input and expressed as fold-enrichment. ***, *p* < 0.001. *C,* chromatin immunoprecipitation using the livers of *Hnf4a*^ΔH^ and *Hnf4a^f/f^* mice with anti-HNF4α antibody and normal goat IgG. The regions between the proximal and the distal HNF4α-binding sites in the mouse *Tfr2* promoter and the regions without HNF4α-binding site in the mouse *Hmgcs2* gene were amplified by real time PCR. The data were normalized relative to the input and expressed as fold-enrichment over data from the IgG control. ***, *p* < 0.001. *D,* 10 nm siRNA for HNF4α (*siHNF4*α) or negative control (*siControl*) was transfected into HepG2 cells. Real time PCR for *HNF4a* (*white bar*) and *TFR2* mRNA (*black bar*) from total RNA of siRNA-transfected cells (*n* = 3) is shown. Data are mean ± S.D. **, *p* < 0.01; ***, *p* < 0.001 compared with siControl-treated cells.

To determine whether HNF4α can directly bind to both HNF4α-binding sites in the *Tfr2* promoter, gel mobility shift analysis was performed (Fig. 5*A*). Nuclear extracts from HepG2 cells bound to the distal (+182/+201) and proximal (+830/+842) HNF4α-binding sites (Fig. 5*A, lane 2*, *lower arrows*). This complex was diminished by the addition of excess amounts of unlabeled *Tfr2* competitor and the *Otc* competitor that contains a *bona fide* HNF4α-binding site (Fig. 5*A, lanes 3* and *4*) (19) but not the competitor that has mutations in the HNF4α-binding site of the *Tfr2* promoter (Fig. 5*A, lane 5*). Moreover, the complex was supershifted by anti-HNF4α antibody (Fig. 5*A, lane 6*, *upper arrow*) but not the unrelated anti-peroxisome proliferator-activated receptor α antibody (*lane 7*). These results indicate that HNF4α indeed binds to the distal and proximal regions of the *Tfr2* promoter. Next, chromatin immunoprecipitation (ChIP) was used to determine whether HNF4α directly binds to the *Tfr2* promoter *in vivo*. HNF4α bound to the predicted HNF4α-binding sites in HepG2 cells as compared with IgG control (Fig. 5*B*). In addition, ChIP analysis using the livers of *Hnf4a^f/f^* and *Hnf4a*^ΔH^ mice indicated that hepatic HNF4α in *Hnf4a^f/f^* mice significantly bound to the promoter region, and the binding was lower in *Hnf4a^f/f^* mouse livers (Fig. 5*C*), suggesting that HNF4α directly and physiologically binds to the promoter region of the *Tfr2* gene in human and mouse liver.

To determine whether regulation of hepatic *Tfr2* expression by HNF4α observed in mice could be also maintained in a human hepatocyte-derived cell line, HNF4α knockdown by siRNAs was performed revealing that HNF4α was suppressed in HepG2 cells (Fig. 5*D*). Expression of *TFR2* mRNA was decreased by HNF4α knockdown, indicating that expression of the *TFR2* mRNA is positively correlated with HNF4α expression in HepG2 cells (Fig. 5*D*).

## Discussion

The present data show that serum iron levels are decreased in *Hnf4a*^ΔH^ mice without significant changes in RBC, HBG, and hematocrit and no evidence for iron deficiency anemia. Changes in the expression of multiple genes in *Hnf4a*^ΔH^ mice make it difficult to ascribe a phenotype to loss of expression of a single gene. However, expression of hepatic *Tf* mRNA and serum TF protein was decreased in *Hnf4a*^ΔH^ mice, due to loss of direct transactivation of *TF* by HNF4α (15). Hypotransferrinemic mice carrying a mutation in the *Tf* gene die from iron deficiency anemia before weaning, but these mice can be rescued by injection of serum or purified TF (26). Thus, the reduced expression of TF would only partially cause hypoferremia in *Hnf4a*^ΔH^ mice. Serum TF protein still remains about 70% of normal as revealed by Western blot data, suggesting that the symptoms in *Hnf4a*^ΔH^ mice would not be severe enough to develop iron deficiency anemia. Because serum TF protein has a relatively long half-life (8–10 days), the majority of serum TF protein may remain in *Hnf4a*^ΔH^ mice despite that TF protein is mainly expressed in the liver, and hepatic expression of *Tf* mRNA in *Hnf4a*^ΔH^ mice was decreased to 20% compared with control mice. Furthermore, expression of *Tfr1* mRNA was markedly increased in *Hnf4a*^ΔH^ mice. *Tfr1* mRNA is post-transcriptionally regulated via iron-response elements in the 3′-untranslated regions by iron-regulatory proteins during low iron levels, resulting in *Tfr1* mRNA stabilization and increased TFR1 protein (3, 28). However, both liver iron content and expression of IRP-1 and -2 remained unchanged in *Hnf4a*^ΔH^ mice, suggesting that TFR1 protein expression would also be increased in *Hnf4a*^ΔH^ mice as well as *Tfr1* mRNA without post-transcriptional regulation. Thus, increased iron uptake into the liver by increased TFR1 may cause the hypoferremia found in *Hnf4a*^ΔH^ mice.

The other cause of hypoferremia in *Hnf4a*^ΔH^ mice might be due to up-regulation of *Hamp* expression. Excess HAMP degrades or internalizes duodenal FPN1 and inhibits iron export into the bloodstream, leading to induction of hypoferremia, and thus the HAMP-FPN1 axis is the most important regulator of iron homeostasis (1, 12). Expression of *HAMP* is positively regulated at the transcriptional level through hemojuvelin, TFR2, and the pro-inflammatory IL6/STAT3 pathway (1, 2, 10, 29). However, despite the decreased expression of *Hjv* and *Tfr2* mRNA, *Hamp* mRNA is elevated 3-fold in *Hnf4a*^ΔH^ mice. In contrast, *Hnf4a*^ΔH^ mice showed hepatosteatosis and increased serum alanine transaminase, an indicator of liver dysfunction and inflammation (18). Moreover, ROS are known to induce inflammation through the production of proinflammatory cytokines (30), and hepatic expression of CAT, SOD1, and GPX1 that degrade ROS was decreased in *Hnf4a*^ΔH^ mice (31). These data indicate that the accumulation of ROS could lead to liver inflammation, and the resultant up-regulation of *Hamp* in *Hnf4a*^ΔH^ mice could be caused by enhanced inflammatory signaling.

Even though reduced expression of hepatic *Tfr2* that causes hereditary hemochromatosis when mutated in humans (7), *Hnf4a*^ΔH^ mice did not show evidence of this disorder. Because hepatic *Hamp* expression in both the complete and liver-specific *Tfr2*-null mice was decreased at a similar age as the *Hnf4a*^ΔH^ mice used in this study (32, 33) and because HAMP is the downstream regulator of TFR2, up-regulation of *Hamp* might be the main reason why *Hnf4a*^ΔH^ mice do not show hemochromatosis despite the down-regulation of *Tfr2* expression. It is noteworthy that liver-specific *Tfr2*-null mice also exhibited elevated transferrin saturation (33), suggesting that absorption of iron from the duodenal FPN1 would be increased by reduced expression of *Hamp*. In contrast, *Hnf4a*^ΔH^ mice exhibited normal transferrin saturation. Thus, the phenotype of *Hnf4a*^ΔH^ mice does not overlap with those of liver-specific *Tfr2*-null mice, despite the fact that HNF4α is an upstream regulator of TFR2. A potential reason for this discrepancy is that lack of HNF4α may have a profound effect on hepatic iron metabolism compared with that of TFR2.

This study further shows that HNF4α binds and activates transcription of *Tfr2*. Previous studies showed that the mouse *Tfr2* proximal and distal promoter was transactivated in the presence of GATA-1 and C/EBPα (27). Of these factors, GATA-1 would not be an essential factor to transactivate *Tfr2* in the liver because GATA-1 is a master regulator for erythroid differentiation and transactivates erythroid-specific genes (34, 35). Moreover, C/EBPα is enriched in the liver and positively regulates many liver-enriched genes (36). Although the expression of hepatic *Cebpa* was decreased by 45% in *Hnf4a*^ΔH^ mice (37), no significant change of hepatic *Tfr2* mRNA expression was detected in *Cebpa*^ΔH^ mice. Additionally, the transactivation potential of C/EBPα alone for the *Tfr2* promoter without GATA-1 is much lower as compared with that with GATA-1 (27), and the contribution of C/EBPα to *Tfr2* regulation would be minor in the liver. Although details on transcriptional regulation of the *Tfr2* gene in the liver are not clear, HNF4α could directly regulate the *Tfr2* expression through at least two HNF4α-binding sites. Unlike the highly conserved binding site at +830/+842, the binding site at +182/+201 is not conserved among the species, and disruption of both binding sites did not completely inhibit the HNF4α-dependent transactivation. However, HNF4α knockdown greatly suppressed *TFR2* expression in human hepatoma cells, indicating that there might be additional HNF4α-binding sites within the proximal promoter. This study revealed that GC-rich regions in the distal promoter were also important for maximal transactivation of the *Tfr2* gene promoter. It was reported that cooperation of the HNF4α and GC-rich regions is important for transactivation of the human *APOC3* gene and mouse *Slc27a5* gene (38, 39), indicating that the GC-rich regions and the HNF4α-binding sites in the *Tfr2* promoter can be the basal *cis*-elements and the liver-specific enhancer elements, respectively.

Although HNF4α was found to directly regulate *Tfr2* expression, the mechanism for repressed expression of hepatic *Hamp* expression in *Hnf4a*^ΔH^ mice could not be determined in this study. Because HNF4α is a master regulator for maintenance of liver-specific functions and severe liver dysfunction and inflammation were actually observed in *Hnf4a*^ΔH^ mice, the possibility cannot be excluded that complicated secondary effects would transactivate *Hamp* expression and lower the effect of reduced expression of *Tfr2* in *Hnf4a*^ΔH^ mice. Despite the fact that *Tfr2* is a direct target for HNF4α, remarkable changes of gene expression override the potential effects and phenotypes caused by decreased expression of *Tfr2* in *Hnf4a*^ΔH^ mice. Consequently, HNF4α is expected to transactivate *Hamp* through modulation of *Tfr2* expression in the normal liver.

In conclusion, hepatic HNF4α significantly contributes to iron homeostasis and directly regulates *Tfr2* expression. Thus, augmentation of hepatic HNF4α signaling could be of value for the treatment of hypoferremia and anemia.

## Author Contributions

S. M., M. O., Y. M., S. S., and T. F. conducted most of the experiments and analyzed the results. M. U. M. conducted experiments of IronChip analysis. M. T. and N. A. conducted experiments of real time PCR and ChIP analysis. H. O. and M. S. conducted experiments of siRNA. Y. I. conceived the idea for the project and wrote the paper with F. J. G.

## Footnotes

2 The abbreviations used are:

ROS: reactive oxygen species
HNF4α: hepatocyte nuclear factor 4α
TUBG: γ-tubulin
TF: transferrin.

## References

[1] Ganz T. (2013) Systemic iron homeostasis. Physiol. Rev. 93, 1721–1741 24137020 10.1152/physrev.00008.2013

[2] Muñoz M., García-Erce J. A., and Remacha A. F. (2011) Disorders of iron metabolism. Part 1: molecular basis of iron homoeostasis. J. Clin. Pathol. 64, 281–286 21177266 10.1136/jcp.2010.079046

[3] Wang J., and Pantopoulos K. (2011) Regulation of cellular iron metabolism. Biochem. J. 434, 365–381 21348856 10.1042/BJ20101825 PMC3048577

[4] Aisen P. (2004) Transferrin receptor 1. Int. J. Biochem. Cell Biol. 36, 2137–2143 15313461 10.1016/j.biocel.2004.02.007

[5] Hayashi A., Wada Y., Suzuki T., and Shimizu A. (1993) Studies on familial hypotransferrinemia: unique clinical course and molecular pathology. Am. J. Hum. Genet. 53, 201–213 8317485 PMC1682235

[6] Kawabata H., Yang R., Hirama T., Vuong P. T., Kawano S., Gombart A. F., and Koeffler H. P. (1999) Molecular cloning of transferrin receptor 2. A new member of the transferrin receptor-like family. J. Biol. Chem. 274, 20826–20832 10409623 10.1074/jbc.274.30.20826

[7] Camaschella C., Roetto A., Calì A., De Gobbi M., Garozzo G., Carella M., Majorano N., Totaro A., and Gasparini P. (2000) The gene TFR2 is mutated in a new type of haemochromatosis mapping to 7q22. Nat. Genet. 25, 14–15 10802645 10.1038/75534

[8] West A. P. Jr., Bennett M. J., Sellers V. M., Andrews N. C., Enns C. A., and Bjorkman P. J. (2000) Comparison of the interactions of transferrin receptor and transferrin receptor 2 with transferrin and the hereditary hemochromatosis protein HFE. J. Biol. Chem. 275, 38135–38138 11027676 10.1074/jbc.C000664200

[9] Worthen C. A., and Enns C. A. (2014) The role of hepatic transferrin receptor 2 in the regulation of iron homeostasis in the body. Front. Pharmacol. 5, 34 24639653 10.3389/fphar.2014.00034 PMC3944196

[10] Gao J., Chen J., Kramer M., Tsukamoto H., Zhang A. S., and Enns C. A. (2009) Interaction of the hereditary hemochromatosis protein HFE with transferrin receptor 2 is required for transferrin-induced hepcidin expression. Cell Metab. 9, 217–227 19254567 10.1016/j.cmet.2009.01.010 PMC2673483

[11] Nemeth E., Tuttle M. S., Powelson J., Vaughn M. B., Donovan A., Ward D. M., Ganz T., and Kaplan J. (2004) Hepcidin regulates cellular iron efflux by binding to ferroportin and inducing its internalization. Science 306, 2090–2093 15514116 10.1126/science.1104742

[12] Yun S., and Vincelette N. D. (2015) Update on iron metabolism and molecular perspective of common genetic and acquired disorder, hemochromatosis. Crit. Rev. Oncol. Hematol. 95, 12–25 25737209 10.1016/j.critrevonc.2015.02.006

[13] Sladek F. M., and Seidel S. D. (2001) in Nuclear Receptor and Genetic Disease (Burris T. P., and McCabe E., eds) pp. 309–361, Academic Press, San Diego

[14] Schrem H., Klempnauer J., and Borlak J. (2002) Liver-enriched transcription factors in liver function and development. Part I: the hepatocyte nuclear factor network and liver-specific gene expression. Pharmacol. Rev. 54, 129–158 11870262 10.1124/pr.54.1.129

[15] Schaeffer E., Guillou F., Part D., and Zakin M. M. (1993) A different combination of transcription factors modulates the expression of the human transferrin promoter in liver and Sertoli cells. J. Biol. Chem. 268, 23399–23408 8226864

[16] Truksa J., Lee P., and Beutler E. (2009) Two BMP responsive elements, STAT, and bZIP/HNF4/COUP motifs of the hepcidin promoter are critical for BMP, SMAD1, and HJV responsiveness. Blood 113, 688–695 18997172 10.1182/blood-2008-05-160184 PMC2628375

[17] Courselaud B., Pigeon C., Inoue Y., Inoue J., Gonzalez F. J., Leroyer P., Gilot D., Boudjema K., Guguen-Guillouzo C., Brissot P., Loréal O., and Ilyin G. (2002) C/EBPα regulates hepatic transcription of hepcidin, an antimicrobial peptide and regulator of iron metabolism. J. Biol. Chem. 277, 41163–41170 12183449 10.1074/jbc.M202653200

[18] Hayhurst G. P., Lee Y. H., Lambert G., Ward J. M., and Gonzalez F. J. (2001) Hepatocyte nuclear factor 4α (nuclear receptor 2A1) is essential for maintenance of hepatic gene expression and lipid homeostasis. Mol. Cell Biol. 21, 1393–1403 11158324 10.1128/MCB.21.4.1393-1403.2001 PMC99591

[19] Inoue Y., Hayhurst G. P., Inoue J., Mori M., and Gonzalez F. J. (2002) Defective ureagenesis in mice carrying a liver-specific disruption of hepatocyte nuclear factor 4α (HNF4α). HNF4α regulates ornithine transcarbamylase *in vivo*. J. Biol. Chem. 277, 25257–25265 11994307 10.1074/jbc.M203126200

[20] Inoue Y., Yu A. M., Yim S. H., Ma X., Krausz K. W., Inoue J., Xiang C. C., Brownstein M. J., Eggertsen G., Björkhem I., and Gonzalez F. J. (2006) Regulation of bile acid biosynthesis by hepatocyte nuclear factor 4α. J. Lipid Res. 47, 215–227 16264197 10.1194/jlr.M500430-JLR200 PMC1413576

[21] Inoue Y., Inoue J., Lambert G., Yim S. H., and Gonzalez F. J. (2004) Disruption of hepatic C/EBPα results in impaired glucose tolerance and age-dependent hepatosteatosis. J. Biol. Chem. 279, 44740–44748 15292250 10.1074/jbc.M405177200

[22] Muckenthaler M., Richter A., Gunkel N., Riedel D., Polycarpou-Schwarz M., Hentze S., Falkenhahn M., Stremmel W., Ansorge W., and Hentze M. W. (2003) Relationships and distinctions in iron-regulatory networks responding to interrelated signals. Blood 101, 3690–3698 12393473 10.1182/blood-2002-07-2140

[23] Muckenthaler M., Roy C. N., Custodio A. O., Miñana B., deGraaf J., Montross L. K., Andrews N. C., and Hentze M. W. (2003) Regulatory defects in liver and intestine implicate abnormal hepcidin and Cybrd1 expression in mouse hemochromatosis. Nat. Genet. 34, 102–107 12704390 10.1038/ng1152

[24] Richter A., Schwager C., Hentze S., Ansorge W., Hentze M. W., and Muckenthaler M. (2002) Comparison of fluorescent tag DNA labeling methods used for expression analysis by DNA microarrays. BioTechniques 33, 620–628 12238772 10.2144/02333rr05

[25] Bartnikas T. B. (2012) Known and potential roles of transferrin in iron biology. Biometals 25, 677–686 22294463 10.1007/s10534-012-9520-3 PMC3595092

[26] Bernstein S. E. (1987) Hereditary hypotransferrinemia with hemosiderosis, a murine disorder resembling human atransferrinemia. J. Lab. Clin. Med. 110, 690–705 3681112

[27] Kawabata H., Germain R. S., Ikezoe T., Tong X., Green E. M., Gombart A. F., and Koeffler H. P. (2001) Regulation of expression of murine transferrin receptor 2. Blood 98, 1949–1954 11535534 10.1182/blood.v98.6.1949

[28] Wallander M. L., Leibold E. A., and Eisenstein R. S. (2006) Molecular control of vertebrate iron homeostasis by iron regulatory proteins. Biochim. Biophys. Acta 1763, 668–689 16872694 10.1016/j.bbamcr.2006.05.004 PMC2291536

[29] Core A. B., Canali S., and Babitt J. L. (2014) Hemojuvelin and bone morphogenetic protein (BMP) signaling in iron homeostasis. Front. Pharmacol. 5, 104 24860505 10.3389/fphar.2014.00104 PMC4026703

[30] Rolo A. P., Teodoro J. S., and Palmeira C. M. (2012) Role of oxidative stress in the pathogenesis of nonalcoholic steatohepatitis. Free Radic. Biol. Med. 52, 59–69 22064361 10.1016/j.freeradbiomed.2011.10.003

[31] Handy D. E., and Loscalzo J. (2012) Redox regulation of mitochondrial function. Antioxid. Redox Signal. 16, 1323–1367 22146081 10.1089/ars.2011.4123 PMC3324814

[32] Wallace D. F., Summerville L., Lusby P. E., and Subramaniam V. N. (2005) First phenotypic description of transferrin receptor 2 knockout mouse, and the role of hepcidin. Gut 54, 980–986 15951546 10.1136/gut.2004.062018 PMC1774629

[33] Wallace D. F., Summerville L., and Subramaniam V. N. (2007) Targeted disruption of the hepatic transferrin receptor 2 gene in mice leads to iron overload. Gastroenterology 132, 301–310 17241880 10.1053/j.gastro.2006.11.028

[34] Pevny L., Simon M. C., Robertson E., Klein W. H., Tsai S. F., D'Agati V., Orkin S. H., and Costantini F. (1991) Erythroid differentiation in chimaeric mice blocked by a targeted mutation in the gene for transcription factor GATA-1. Nature 349, 257–260 1987478 10.1038/349257a0

[35] Welch J. J., Watts J. A., Vakoc C. R., Yao Y., Wang H., Hardison R. C., Blobel G. A., Chodosh L. A., and Weiss M. J. (2004) Global regulation of erythroid gene expression by transcription factor GATA-1. Blood 104, 3136–3147 15297311 10.1182/blood-2004-04-1603

[36] Schrem H., Klempnauer J., and Borlak J. (2004) Liver-enriched transcription factors in liver function and development. Part II: the C/EBPs and D site-binding protein in cell cycle control, carcinogenesis, circadian gene regulation, liver regeneration, apoptosis, and liver-specific gene regulation. Pharmacol. Rev. 56, 291–330 15169930 10.1124/pr.56.2.5

[37] Wiwi C. A., Gupte M., and Waxman D. J. (2004) Sexually dimorphic P450 gene expression in liver-specific hepatocyte nuclear factor 4α-deficient mice. Mol. Endocrinol. 18, 1975–1987 15155787 10.1210/me.2004-0129

[38] Kardassis D., Falvey E., Tsantili P., Hadzopoulou-Cladaras M., and Zannis V. (2002) Direct physical interactions between HNF-4 and Sp1 mediate synergistic transactivation of the apolipoprotein CIII promoter. Biochemistry 41, 1217–1228 11802721 10.1021/bi015618f

[39] Inoue Y., Yu A. M., Inoue J., and Gonzalez F. J. (2004) Hepatocyte nuclear factor 4α is a central regulator of bile acid conjugation. J. Biol. Chem. 279, 2480–2489 14583614 10.1074/jbc.M311015200

